# Reduced right/left ventricular blood pool T2 ratio on cardiac magnetic resonance indicates cognitive impairment in heart failure secondary to ischemic heart disease

**DOI:** 10.1186/s12880-026-02359-3

**Published:** 2026-04-22

**Authors:** Yadong Cui, Chong Zheng, Shanshan Gu, Jin Si, Keling Xiao, Yujie Hu, Yang Yang, Jing Li, Jie Lu

**Affiliations:** 1 Department of Radiology and Nuclear Medicine, Xuanwu Hospital, Capital Medical University, No.45 Changchun Street, Xicheng District, Beijing, 100053 China; 2 Beijing Key Laboratory of Magnetic Resonance Imaging and Brain Informatics, Beijing, China; 3 Department of Geriatrics, National Clinical Research Center for Geriatric Diseases, Xuanwu Hospital Capital Medical University, Beijing, China; 4 Central Research Institute, United Imaging Healthcare Group, Shanghai, China; 5 Beijing United Imaging Research Institute of Intelligent Imaging, Beijing, China

**Keywords:** Heart failure, Cardiac magnetic resonance, T2 mapping, Cognitive impairment

## Abstract

**Purpose:**

The right-to-left ventricular blood pool T2 ratio (RV/LV T2 ratio) derived from cardiac magnetic resonance (CMR) T2 mapping is a potential biomarker of blood oxygenation. This study investigated the association between RV/LV T2 ratio and cognitive performance in heart failure (HF) secondary to ischemic heart disease (IHD).

**Methods:**

This retrospective study included 52 patients with chronic HF and 26 healthy controls, all of whom underwent CMR and neuropsychological testing. Regions of interest were manually drawn in RV and LV blood pools to calculate RV/LV T2 ratio. Cognitive performance was assessed by the Mini-Mental State Examination (MMSE) and Montreal Cognitive Assessment (MoCA). Group comparisons and linear, multivariate, and mediation analyses were performed.

**Results:**

HC demonstrated higher RV/LV T2 ratio than patients with HF with preserved or reduced and mildly reduced ejection fraction (*p* = 0.012, and *p* < 0.001). Patients with cognitive impairment (MoCA-defined) showed lower RV/LV T2 ratios than cognitively normal patients (*p* = 0.010). RV/LV T2 ratio was positively related to MMSE (β = 0.336, *p* = 0.020) and MoCA score (β = 0.563, *p* < 0.001). In multivariate linear regression analysis, RV/LV T2 ratio was the only CMR predictor for MoCA score (β = 0.340, *p* = 0.015). Mediation analysis showed RV/LV T2 ratio partially mediated the correlation between stroke volume and MoCA score, with a mediation effect ratio of 44.3%.

**Conclusion:**

RV/LV T2 ratio is an additional CMR biomarker to evaluate the cognitive performance in patients with HF secondary to IHD.

## Introduction

Heart failure (HF) is a complicated pathological condition characterized by symptoms resulting from impairment of cardiac structure and function. It remains a leading cause of hospitalization and mortality among the elderly population [1]. Accumulating evidence indicates that patients with HF are at increased risk of cognitive impairment, which is associated with poor clinical outcomes [2, 3]. The underlying mechanisms are multifactorial, and not yet fully elucidated, potentially involving systemic hypoperfusion, inflammatory response, and oxidative stress [4]. Reduced cerebral blood flow (CBF) due to low cardiac output is considered as a major mechanism. Impaired CBF would affect multiple brain regions critical for memory, executive function, and language [5]. Chronic HF patients with reduced systemic perfusion usually experience impaired oxygen delivery to peripheral target tissues and circulatory hypoxia [6]. Chronic cerebral hypoperfusion may result in hypoxic injury, triggering various pathophysiological changes in the brain, such as disruption of the blood-brain barrier (BBB) and impaired clearance of neurotoxic substances [4]. Inflammation and oxidative stress also contribute to neuroinflammation and neuronal damage in HF by disrupting the BBB and activating glial cells, ultimately leading to cognitive impairment [7].

Cardiac magnetic resonance (CMR) is a widely available and reliable imaging modality for assessing cardiac structure, function, and tissue characterization, playing an essential in the evaluation and management of HF patients [8–10]. Parametric mapping techniques enable noninvasive quantification of various myocardial pathological changes through tissue magnetic properties and have become an integral component of comprehensive CMR protocol [11]. Among these, T2 mapping allows for in vivo detection of myocardial edema and represents a valuable tool in the diagnosis, treatment, and prognosis of various myocardial diseases [12–14]. In addition to myocardial tissue, T2 mapping is sensitive to changes in the concentrations of oxyhemoglobin and deoxyhemoglobin and has been used to measure the blood oxygenation level [15, 16]. However, previous methods required additional sequence, excess scan time, and complex post-processing approach, which prohibited its clinical application. Recently, right/left ventricular blood pool T2 ratio (RV/LV T2 ratio) has been proposed as a promising new CMR biomarker about the blood oxygen saturation difference between RV and LV and initially applied to several cardiovascular diseases such as left-to-right shunts [17] and pulmonary hypertension [18, 19]. Additionally, this method has also been reported to correlate with the exercise capacity in patients with chronic HF, suggesting its potential role for early detection of silent hypoxia [20].

Emerging studies have demonstrated the value of the RV/LV T2 ratio for risk stratification of HF, and prognostic prediction of congestive HF after ST-segment elevation myocardial infarction (STEMI) [21, 22]. However, the correlation between this ratio and cognitive impairment in patients with HF has not been investigated. Therefore, we postulate RV/LV T2 ratio is correlated with the degree of cognitive impairment in HF. As ischemic heart disease (IHD) is among the leading etiologies of HF, we aimed to investigate the relationship between RV/LV T2 ratio and cognitive performance in HF patients secondary to IHD, and to further explore whether RV/LV T2 ratio mediates the association between cardiac dysfunction and cognitive impairment.

## Materials and methods

### Study population

Fifty-eight HF patients and 26 sex- and age-matched healthy controls (HC) who underwent CMR examination and cognitive assessment between September 2020 and November 2021 were retrospectively enrolled from our hospital. This study was approved by the institutional review board and written informed consent was obtained. Inclusion criteria for HF patients were as follows: age > 18 years; diagnosis of coronary artery disease confirmed by coronary angiography; diagnosis of chronic HF according to the European Society of Cardiology guideline [23]; successfully completed cognitive assessment. Exclusion criteria: patients with cerebral diseases affecting cognitive performance: stroke, severe white matter hyperintensity (Fazekas score = 3) or lacunar infarcts, benign or malignant tumors, and psychiatric diseases; patients with severe valvular diseases, atrial fibrillation, and hypertrophic cardiomyopathy; poor imaging quality. Patients classified as New York Heart Association (NYHA) class IV were excluded due to safety and imaging quality concerns. Finally, 52 patients were included for further analysis. According to the left ventricular ejection fraction (LVEF), patients were classified as heart failure with reduced and mildly reduced ejection fraction (HFrEF/HFmrEF) (LVEF < 50%) and heart failure with preserved ejection fraction (HFpEF) (LVEF ≥ 50%) [1].

### Neuropsychological assessment

Given the lack of consensus on the formal screening tools used for assessing cognitive impairment in patients with HF, we employed the widely recognized neuropsychological tests, Chinese revised version of Mini-Mental State Examination (MMSE) and Montreal Cognitive Assessment (MoCA) (Beijng version), to evaluate patients’ global cognitive performance on the same day of CMR examination [24]. Neuropsychological assessors (J. S. and K. X.) were blinded to the CMR results. MMSE score < 24 or MoCA score < 26 was diagnosed as cognitively impaired.

### CMR imaging

CMR images were acquired on a 3.0-Tesla uPMR 790 system (United Imaging Healthcare, Shanghai, China) using a 12-element phase-array cardiac coil. Imaging was electrocardiogram (ECG) gated and acquired in end-expiratory breath hold. The CMR imaging protocol consisted of cine, T2 mapping, and late gadolinium enhancement (LGE). Balanced steady-state free precession technique was acquired in long-axis and short-axis slices for cine imaging with the following parameters: field of view (FOV) 36 cm × 32 cm, matrix 288 × 256, repetition time (TR)/ echo time (TE) 3.1/1.4 ms, slice thickness 8 mm, slice gap 2 mm, 25 images per cardiac cycle. T2 mapping was obtained at end-diastole on three short-axis slices. T2-prepared single-shot balanced steady-state free precession sequence was performed for T2 mapping acquisition with the following parameters: FOV 36 cm × 32 cm, matrix 288 × 229, TR/TE 3.0/1.4 ms, slice thickness 8 mm, preparation times 0/30/55 ms. Ten minutes after injection of 0.15 mmol/kg contrast agent, LGE images were obtained using phase-sensitive inversion recovery (PSIR) sequence with the same slice location and thickness to cine images. Imaging parameters: FOV 36 cm × 36 cm, matrix 240 × 180, TR/TE 4.7/1.9 ms, inversion time 300–330 ms for nulling of remote normal myocardium.

### Imaging analysis

**Fig. 1:**
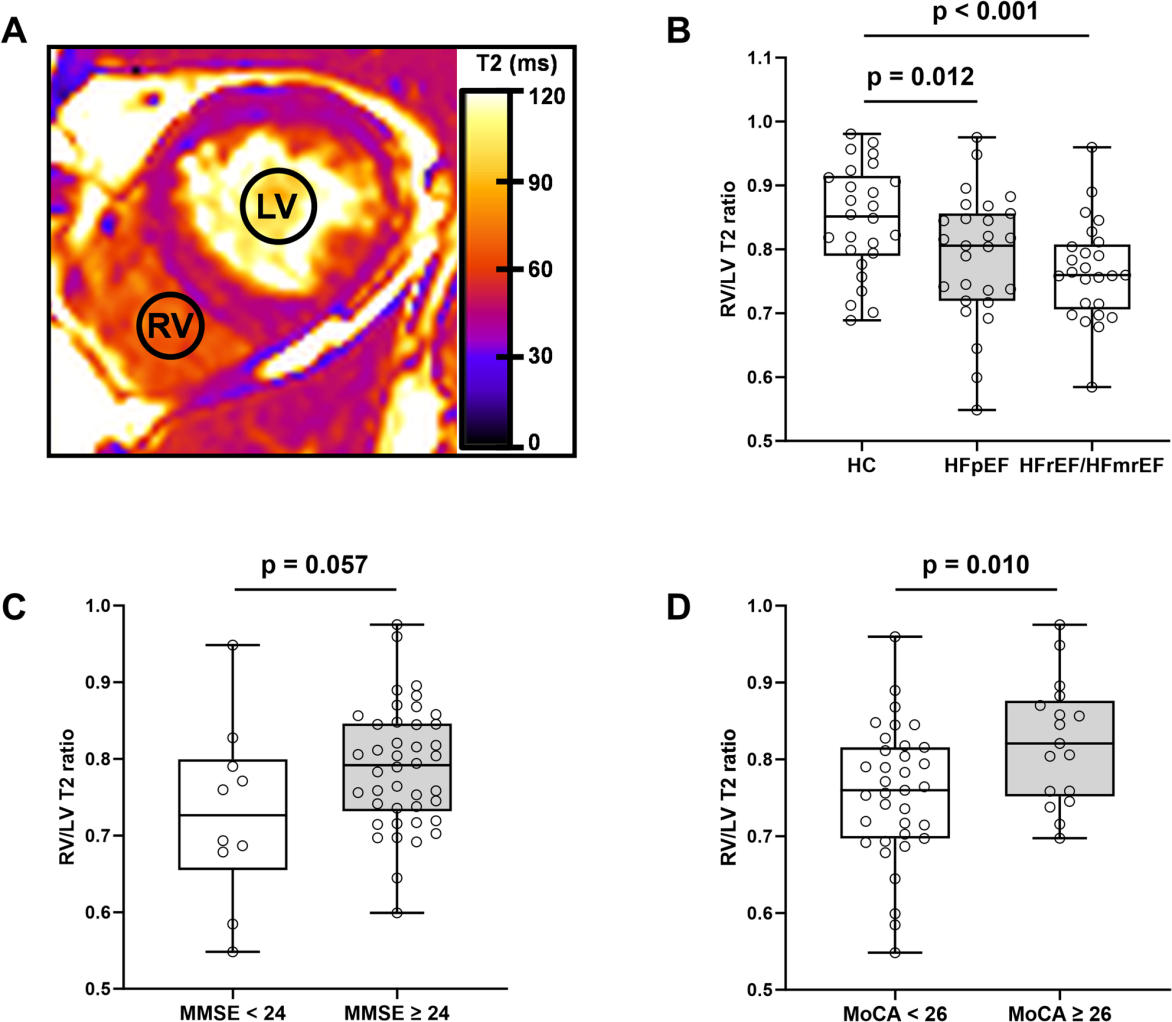
Measurement of T2 value of RV and LV blood pool on short-axis T2 map image (**A**). Boxplots show comparisons of RV/LV T2 ratio between HFpEF and HFrEF/HFmrEF (**B**), normal and impaired cognition based on MMSE score (**C**) and MoCA score (**D**). RV, right ventricle; LV, left ventricle; HFpEF, heart failure with preserved ejection fraction; HFrEF/HFmrEF, heart failure with reduced and mildly reduced ejection fraction; MMSE, Mini-Mental State Exam; MoCA, Montreal Cognitive Assessment

Results of CMR parameters were obtained using cvi42 (Circle Cardiovascular Imaging Inc., Calgary, Alberta, Canada). Imaging analysis was performed blinded to patients’ clinical information and cognitive assessment results. Left ventricular function parameters were obtained by semi-automatically defining the endocardial and epicardial contours on short-axis cine images. Pixel-wise T2 maps were automatically generated from raw data with motion correction on scanner. Two circular regions of interest (ROI) ≥ 1 cm^2^ were manually placed on T2 maps from basal, mid-ventricular, and apical short-axis slices to measure the mean transverse relaxation times of RV and LV blood pool, avoiding obvious flow artifacts and excluding papillary muscle and myocardium (Fig. 1A). The average RV/LV T2 ratio from three short-axis planes was used as the final result. Measurements were conducted by two independent observers (Y. C. and C. Z.) with 10 and 6 years of CMR expertise to assess the repeatability of RV/LV T2 ratio. Analysis was repeated by one observer (Y. C.) 1 month after the first measurement. Scar volume was quantified by manually delineating the endocardial and epicardial contours and signal intensity ≥ 5 standard deviations over remote normal myocardium indicates myocardial scar [25].

### Statistical analysis

Statistical analyses were performed using SPSS 27.0 (IBM Corp, Armonk, New York, USA) and MedCalc 20.100 (MedCalc Software Ltd, Ostend, Belgium). Categorical variables were presented as frequency (percentage) and continuous variables were expressed as mean ± standard deviation or median (Q1, Q3). A one-way analysis of variance (ANOVA) test with Bonferroni correction or Kruskal-Wallis test was performed to determine the differences of demographics and CMR parameters between HC and HF subgroups. Differences in demographics and CMR parameters between different HF subtypes and cognitive conditions were compared using chi-square test, Student t test or Mann-Whitney U test where appropriate.

Univariate linear regression analysis was performed to investigate the relationships of cognitive function with demographics and CMR parameters in patients with HF. Variables with statistically significant associations were included in further multivariate regression analysis. Variance inflation factor ≤ 10 indicated the absence of multicollinearity in multivariable analysis. Pearson and Spearman correlation coefficients were performed to analyze the associations between RV/LV T2 ratio and other CMR parameters. Mediation analysis was conducted to analyze the possible mediation effect of RV/LV T2 ratio on the correlation between cardiac function and cognitive function.

The reproducibility of RV/LV T2 ratio measurements in the HF patients were assessed using the intraclass correlation coefficient (ICC) and the Bland–Altman plot. ICC was classified as excellent agreement > 0.9; good agreement 0.75–0.9; moderate agreement 0.5–0.75; and poor agreement < 0.5. Two-tailed *p* value < 0.05 indicated statistically significant.

## Results

### Demographics and CMR parameters in HC and HF patients

A total of 52 HF patients (mean age 61.65 years, 77% male), including 27 HFpEF (mean age 61.92 years, 74% male) and 25 HFrEF/HFmrEF (mean age 61.36 years, 80% male) patients, were included in this study. Demographics and CMR parameters in HC and HF subgroups are summarized in Table 1. HC demonstrated significantly lower HR than patients with HFpEF and HFrEF/HFmrEF (*p* = 0.006). MMSE and MoCA scores of HC were significantly higher than HFpEF and HFrEF/HFmrEF (MMSE: *p* = 0.002, < 0.001; MoCA: *p* < 0.001). Patients with HFrEF/HFmrEF demonstrated significantly higher NYHA class and higher level of N-terminal pro-brain natriuretic peptide (NT-proBNP) than patients with HFpEF (*p* < 0.001, *p* = 0.041). There were no significant differences in other demographic variables between HFpEF and HFrEF/HFmrEF (*p* ≥ 0.407).

**Table 1:** Demographics and CMR parameters in HC and HF patients

Variables	HC (*n* = 26)	HFpEF (*n* = 27)	HFrEF/HFmrEF (*n* = 25)
**Demographics**			
Age, years	61.50 ± 10.6	61.92 ± 10.41	61.36 ± 11.52
Male	20 (77%)	20 (74%)	20 (80%)
Education, years	11.73 ± 2.52	11.18 ± 2.61	11.18 ± 2.93
BMI, kg/m^2^	23.38 ± 4.70	25.15 ± 3.24	24.86 ± 2.14
HR, bpm	74.12 ± 12.82	80.85 ± 14.56	76.67 ± 13.04
SBP, mmHg	125.43 ± 15.76	124.00 ± 20.73	122.54 ± 11.74
DBP, mmHg	70.69 ± 10.67	71.37 ± 11.71	71.54 ± 11.93
Hypertension	0	15 (56%)	14 (56%)
Diabetes mellitus	0	11 (41%)	13 (52%)
Dyslipidemia	0	4 (15%)	7 (28%)
Smoking	0	16 (59%)	15 (60%)
MMSE	29.50 ± 0.65	27.15 ± 2.54	26.12 ± 3.38
MoCA	28.85 ± 0.83	22.81 ± 4.33	22.28 ± 3.75
NYHA class Ⅰ/Ⅱ/Ⅲ	NA	23/3/1	8/9/8
NT-proBNP	NA	303.00 (90.55, 1086.25)	857.00 (289.00, 2832.50)
**CMR**			
EDV, mL	127.97 ± 23.73	125.14 ± 29.09	188.92 ± 53.66
ESV, mL	53.50 ± 14.39	48.51 ± 17.35	127.19 ± 53.30
SV, mL	74.47 ± 10.91	76.63 ± 18.05	61.72 ± 18.93
EDVI, mL/m^2^	79.72 ± 12.89	69.94 ± 12.09	101.31 ± 30.99
ESVI, mL/m^2^	33.24 ± 7.92	27.03 ± 8.30	72.85 ± 32.19
SVI, mL/m^2^	46.48 ± 6.14	42.91 ± 8.31	34.68 ± 10.18
EF, %	58.64 ± 4.31	61.69 ± 8.78	34.44 ± 10.99
CO, L/min	5.08 ± 0.82	4.79 ± 1.14	4.28 ± 1.22
CI, L/min/m^2^	3.16 ± 0.43	2.68 ± 0.53	2.43 ± 0.73
LV mass, g	78.75 ± 20.65	93.02 ± 22.18	113.78 ± 26.19
RV/LV T2 ratio	0.86 ± 0.08	0.78 ± 0.10	0.76 ± 0.08
Scar volume, mL	0	11.92 ± 8.67	33.26 ± 10.44

Abbreviations: HC, healthy controls; HF, heart failure; HFpEF, heart failure with preserved ejection fraction; HFrEF/HFmrEF, heart failure with reduced and mildly reduced ejection fraction; BMI, body mass index; HR, heart rate; SBP, systolic blood pressure; DBP, diastolic blood pressure; MMSE, Mini-Mental State Exam; MoCA, Montreal Cognitive Assessment; NYHA, New York Heart Association; NT-proBNP, N-terminal pro-brain natriuretic peptide; EDV, end-diastolic volume; ESV, end-systolic volume; SV, stroke volume; EDVI, end-diastolic volume index; ESVI, end-systolic volume index; SVI, stroke volume index; EF, ejection fraction; CO, cardiac output; CI, cardiac index; LV, left ventricle; RV, right ventricle

For CMR parameters, HC, and patients with HFpEF showed significantly higher levels of EDV, ESV, SV, EDVI, ESVI, SVI, EF, LV mass than patients with HFrEF/HFmrEF (*p* ≤ 0.02). These parameters were not significantly different between HC and patients with HFpEF (*p* ≥ 0.082). HC demonstrated higher levels of CI and RV/LV T2 ratio than patients with HFpEF and HFrEF/HFmrEF (CI: *p* = 0.010, < 0.001; RV/LV T2 ratio: *p* = 0.012, < 0.001) (Fig. 1B). CO was significantly higher in HC than HFrEF/HFmrEF (*p* = 0.030).

### Demographics and CMR parameters in HF patients with different cognitive performance

Table 2 presents demographics and CMR parameters in patients with normal and impaired cognition. Based on the MMSE score, demographical variables were not significantly different between cognitively normal and impaired HF patients, except for NYHA class (*p* = 0.017). While cognitively impaired patients showed lower level of stroke volume index (SVI) than patients with normal cognition (33.22 ± 9.78 vs. 40.32 ± 9.74 mL/m^2^, *p* = 0.044). According to the MoCA score, cognitively impaired patients were older (65.74 ± 8.65 vs. 53.23 ± 10.23 years, *p* < 0.001), had higher level of NYHA class (*p* = 0.006) and lower level of education (10.34 ± 2.35 vs. 13.29 ± 2.52 years, *p* < 0.001), BMI (24.48 ± 2.69 vs. 26.09 ± 2.59 kg/m^2^, *p* = 0.046), stroke volume (SV) (63.64 ± 18.56 vs. 81.46 ± 16.95 mL, *p* = 0.002), SVI (36.60 ± 10.10 vs. 43.79 ± 8.29 mL/m^2^, *p* = 0.014), and CO (4.28 ± 1.28 vs. 5.09 ± 0.77 L/min, *p* = 0.020). The difference of RV/LV T2 ratio between cognitively impaired and normal HF patients was not significant based on MMSE score (0.73 ± 0.11 vs. 0.79 ± 0.08, *p* = 0.057) (Fig. 1C), while it was significant according to MoCA score (0.75 ± 0.08 vs. 0.82 ± 0.08, *p* = 0.010) (Fig. 1D).

**Table 2:** Demographics and CMR parameters in HF patients with normal and impaired cognition

Variables	MMSE < 24 (*n* = 10)	MMSE ≥ 24 (*n* = 42)	*p* value	MoCA < 26 (*n* = 35)	MoCA ≥ 26 (*n* = 17)	*p* value
**Demographics**						
Age, years	65.60 ± 11.16	60.71 ± 10.70	0.204	65.74 ± 8.65	53.23 ± 10.23	**< 0.001**
Male	7 (70%)	33 (79%)	0.563	26 (74%)	14 (82%)	0.517
Education (years)	10.80 ± 2.09	11.43 ± 2.91	0.523	10.34 ± 2.35	13.29 ± 2.52	**< 0.001**
BMI, kg/m^2^	25.61 ± 2.19	24.87 ± 2.86	0.456	24.48 ± 2.69	26.09 ± 2.59	**0.046**
HR, bpm	78.55 ± 12.44	78.95 ± 14.31	0.939	77.32 ± 13.38	82.00 ± 14.76	0.261
SBP, mmHg	119.11 ± 11.33	124.21 ± 17.91	0.418	123.67 ± 17.60	122.59 ± 16.05	0.835
DBP, mmHg	65.11 ± 7.54	72.81 ± 12.04	0.073	70.38 ± 10.64	73.59 ± 13.66	0.361
Hypertension	4 (40%)	26 (62%)	0.208	20 (57%)	10 (59%)	0.908
Diabetes mellitus	4 (40%)	21 (50%)	0.569	18 (51%)	7 (41%)	0.488
Dyslipidemia	4 (40%)	8 (19%)	0.158	8 (23%)	4 (24%)	0.957
Smoking	4 (40%)	27 (64%)	0.160	22 (63%)	9 (53%)	0.494
NYHA class Ⅰ/Ⅱ/Ⅲ	2/4/4	29/8/5	**0.017**	16/11/8	15/1/1	**0.006**
NT-proBNP	1600.5 (95.87, 3004.75)	445.00 (137.50, 2043.50)	0.610	666.00 (152.00, 2623.00)	325.50 (115.67, 970.75)	0.327
**CMR**						
EDV, mL	166.22 ± 66.91	153.33 ± 49.95	0.496	153.19 ± 57.22	161.18 ± 44.62	0.616
ESV, mL	107.07 ± 63.13	81.41 ± 53.01	0.191	89.56 ± 60.20	79.71 ± 44.75	0.553
SV, mL	59.15 ± 20.05	71.92 ± 19.14	0.066	63.64 ± 18.56	81.46 ± 16.95	**0.002**
EDVI, mL/m^2^	81.47 ± 34.34	85.86 ± 26.53	0.659	84.46 ± 31.28	86.17 ± 19.86	0.838
ESVI, mL/m^2^	63.83 ± 41.49	45.54 ± 29.52	0.111	52.30 ± 36.39	42.38 ± 22.01	0.307
SVI, mL/m^2^	33.22 ± 9.78	40.32 ± 9.74	**0.044**	36.60 ± 10.10	43.79 ± 8.29	**0.014**
EF, %	39.42 ± 15.36	50.77 ± 16.66	0.055	46.42 ± 18.00	53.05 ± 13.73	0.187
CO, L/min	4.02 ± 1.44	4.67 ± 1.11	0.124	4.28 ± 1.28	5.09 ± 0.77	**0.020**
CI, L/min/m^2^	2.28 ± 0.80	2.62 ± 0.58	0.123	2.47 ± 0.71	2.75 ± 0.42	0.140
LV mass, g	100.42 ± 27.32	103.61 ± 26.15	0.733	100.37 ± 26.28	108.40 ± 25.77	0.304
RV/LV T2 ratio	0.73 ± 0.11	0.79 ± 0.08	0.057	0.75 ± 0.08	0.82 ± 0.08	**0.010**
Scar volume, mL	23.07 ± 10.87	21.70 ± 14.94	0.819	22.66 ± 14.58	20.57 ± 14.16	0.637

Abbreviations: CMR, cardiac magnetic resonance; HF, heart failure; MMSE, Mini-Mental State Exam; MoCA, Montreal Cognitive Assessment; BMI, body mass index; HR, heart rate; SBP, systolic blood pressure; DBP, diastolic blood pressure; NYHA, New York Heart Association; NT-proBNP, N-terminal pro-brain natriuretic peptide; EDV, end-diastolic volume; ESV, end-systolic volume; SV, stroke volume; EDVI, end-diastolic volume index; ESVI, end-systolic volume index; SVI, stroke volume index; EF, ejection fraction; CO, cardiac output; CI, cardiac index; LV, left ventricle; RV, right ventricle

### Relationships of cognitive performance with demographics and CMR parameters

**Fig. 2:**
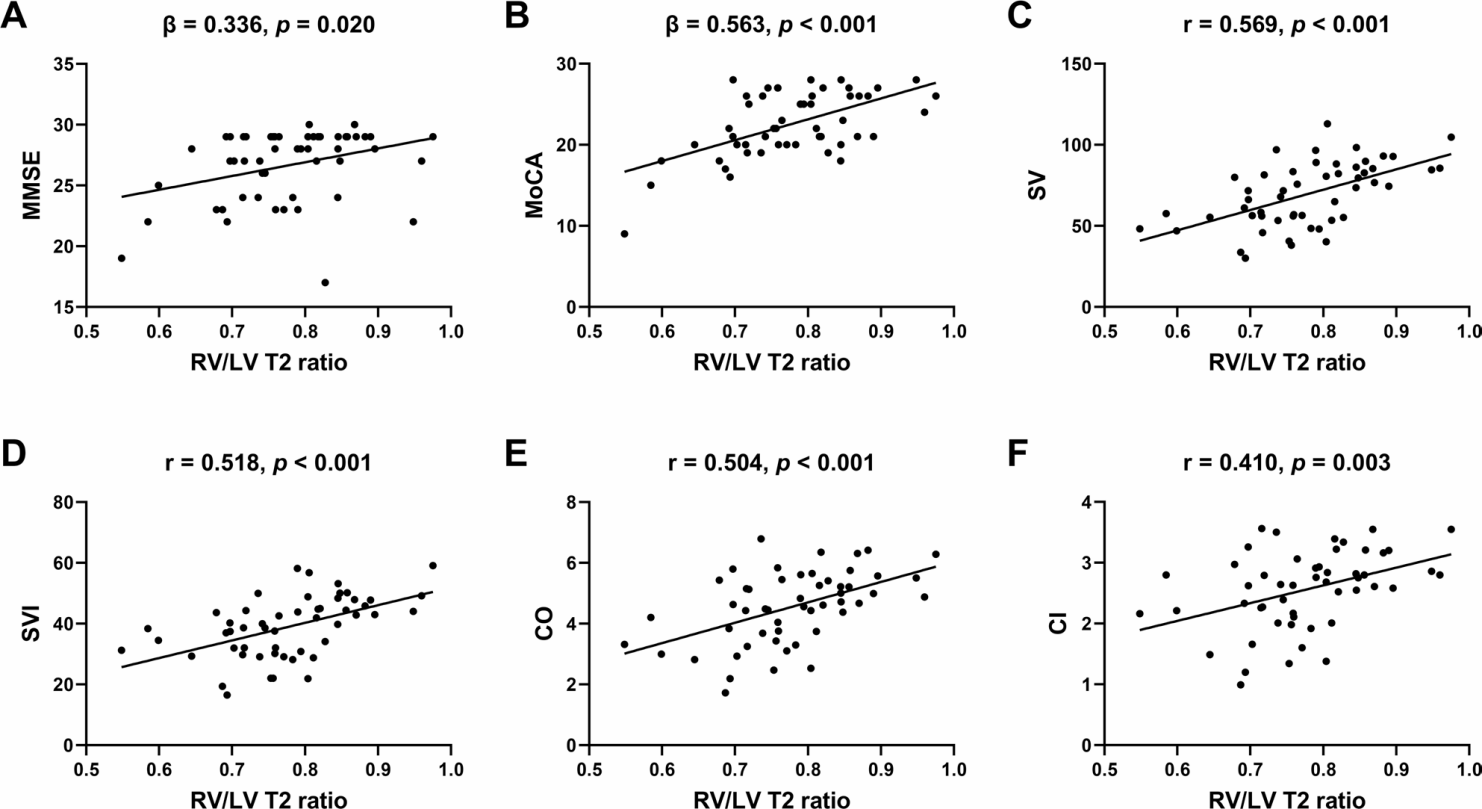
Scatter plots show correlation of RV/LV T2 ratio with MMSE score (**A**), MoCA score (**B**), SV (**C**), SVI (**D**), CO (**E**), and CI (**F**). RV, right ventricle; LV, left ventricle; MMSE, Mini-Mental State Exam; MoCA, Montreal Cognitive Assessment; SV, stroke volume; SVI, stroke volume index; CO, cardiac output; CI, cardiac index

Relationships of MMSE and MoCA score with demographics and CMR parameters in HF patients are presented in Tables 3 and 4. Univariate regression analysis demonstrated that MMSE score was inversely correlated with age (standardized coefficient β = -0.334, *p* = 0.019) and NYHA class (β = -0.352, *p* = 0.011), and was positively related to diastolic blood pressure (DBP) (β = 0.285, *p* = 0.049), SV (β = 0.368, *p* = 0.010), and SVI (β = 0.340, *p* = 0.018). MoCA score was inversely correlated with age (β = -0.509, *p* < 0.001) and NYHA class (β = -0.400, *p* = 0.003), and was positively related to SV (β = 0.503, *p* < 0.001), SVI (β = 0.398, *p* = 0.005), and CO (β = 0.384, *p* = 0.007). RV/LV T2 ratio was positively correlated with MMSE score (β = 0.336, *p* = 0.020) and MoCA score (β = 0.563, *p* < 0.001) (Fig. 2A, B).

**Table 3:** Univariate and multivariate regression linear analyses of MMSE score with demographics and CMR parameters

Variables	Univariate analyses				Multivariate analyses			
	B	SE	β	*p* value	B	SE	β	*p* value
**Demographics**								
Age, years	-0.092	0.038	-0.334	**0.019**	-0.033	0.041	-0.119	0.426
BMI, kg/m^2^	-0.002	0.158	-0.002	0.989				
HR, bpm	0.021	0.031	0.099	0.505				
SBP, mmHg	0.025	0.026	0.141	0.339				
DBP, mmHg	0.072	0.036	0.285	**0.049**	0.074	0.035	0.288	**0.041**
Hypertension	1.392	0.860	0.232	0.112				
Diabetes mellitus	0.661	0.874	0.111	0.756				
Dyslipidemia	-1.600	1.016	-0.226	0.122				
Smoking	0.664	0.884	0.110	0.456				
NYHA class	-1.356	0.510	-0.352	**0.011**	-0.682	0.603	-0.177	0.264
NT-proBNP	0.000	0.000	-0.069	0.654				
**CMR**								
EDV, mL	0.007	0.080	0.126	0.864				
ESV, mL	0.001	0.080	-0.005	0.971				
SV, mL	0.057	0.021	0.368	**0.010**	-0.003	0.060	0.018	0.964
EDVI, mL/m^2^	0.021	0.015	0.203	0.167				
ESVI, mL/m^2^	-0.005	0.013	-0.050	0.735				
SVI, mL/m^2^	0.104	0.042	0.340	**0.018**	0.045	0.119	0.151	0.709
EF, %	0.023	0.026	0.132	0.373				
CO, L/min	0.644	0.385	0.239	0.101				
CI, L/min/m^2^	0.861	0.723	0.173	0.239				
LV mass, g	0.020	0.017	0.167	0.252				
RV/LV T2 ratio	11.039	4.566	0.336	**0.020**	4.755	5.347	0.143	0.379
Scar volume, mL	0.189	0.043	0.199	0.201				

Abbreviations: MMSE, Mini-Mental State Exam; CMR, cardiac magnetic resonance; B, unstandardized coefficient; SE, standard error; β, standardized coefficient; BMI, body mass index; HR, heart rate; SBP, systolic blood pressure; DBP, diastolic blood pressure; NYHA, New York Heart Association; NT-proBNP, N-terminal pro-brain natriuretic peptide; EDV, end-diastolic volume; ESV, end-systolic volume; SV, stroke volume; EDVI, end-diastolic volume index; ESVI, end-systolic volume index; SVI, stroke volume index; EF, ejection fraction; CO, cardiac output; CI, cardiac index; LV, left ventricle; RV, right ventricle

**Table 4:** Univariate and multivariate regression linear analyses of MoCA score with demographics and CMR parameters

Variables	Univariate analyses				Multivariate analyses			
	B	SE	β	*p* value	B	SE	β	*p* value
**Demographics**								
Age, years	-0.190	0.047	-0.509	**< 0.001**	-0.100	0.045	-0.268	**0.032**
BMI, kg/m^2^	0.402	0.206	0.277	0.057				
HR, bpm	-0.003	0.043	-0.010	0.946				
SBP, mmHg	0.001	0.035	-0.001	0.995				
DBP, mmHg	0.049	0.050	0.144	0.330				
Hypertension	1.963	1.165	0.241	0.099				
Diabetes mellitus	-0.248	1.194	-0.031	0.836				
Dyslipidemia	0.651	1.413	0.068	0.647				
Smoking	-0.050	1.207	-0.006	0.967				
NYHA class	-2.076	0.674	-0.400	**0.003**	-1.089	0.671	-0.210	0.112
NT-proBNP	0.000	0.000	-0.133	0.384				
**CMR**								
EDV, mL	0.016	0.011	0.215	0.142				
ESV, mL	0.002	0.011	0.033	0.823				
SV, mL	0.106	0.027	0.503	**< 0.001**	0.130	0.073	0.638	0.082
EDVI, mL/m^2^	0.032	0.021	0.222	0.129				
ESVI, mL/m^2^	-0.005	0.018	-0.045	0.763				
SVI, mL/m^2^	0.165	0.056	0.398	**0.005**	-0.192	0.130	-0.478	0.148
EF, %	0.024	0.035	0.101	0.497				
CO, L/min	1.401	0.497	0.384	**0.007**	-0.425	0.646	-0.126	0.514
CI, L/min/m^2^	1.557	0.969	0.230	0.115				
LV mass, g	0.037	0.023	0.232	0.112				
RV/LV T2 ratio	25.139	5.440	0.563	**< 0.001**	15.254	6.026	0.340	**0.015**
Scar volume, mL	0.189	0.043	0.138	0.201				

Abbreviations: MoCA, Montreal Cognitive Assessment; CMR, cardiac magnetic resonance; B, unstandardized coefficient; SE, standard error; β, standardized coefficient; BMI, body mass index; HR, heart rate; SBP, systolic blood pressure; DBP, diastolic blood pressure; NYHA, New York Heart Association; NT-proBNP, N-terminal pro-brain natriuretic peptide; EDV, end-diastolic volume; ESV, end-systolic volume; SV, stroke volume; EDVI, end-diastolic volume index; ESVI, end-systolic volume index; SVI, stroke volume index; EF, ejection fraction; CO, cardiac output; CI, cardiac index; LV, left ventricle; RV, right ventricle

Multivariate linear regression analysis revealed that DBP independently correlated with MMSE score (β = 0.288, *p* = 0.041), while age was an independent predictor for MoCA score (β = -0.268, *p* = 0.032). RV/LV T2 ratio was the only CMR parameter and strongest predictor for MoCA score (β = 0.340, *p* = 0.015). Besides, SV (β = 0.313, *p* = 0.029) and RV/LV T2 ratio (β = 0.437, *p* = 0.003) remained correlated with MoCA score adjusting for age, sex, and education.

**Fig. 3:**
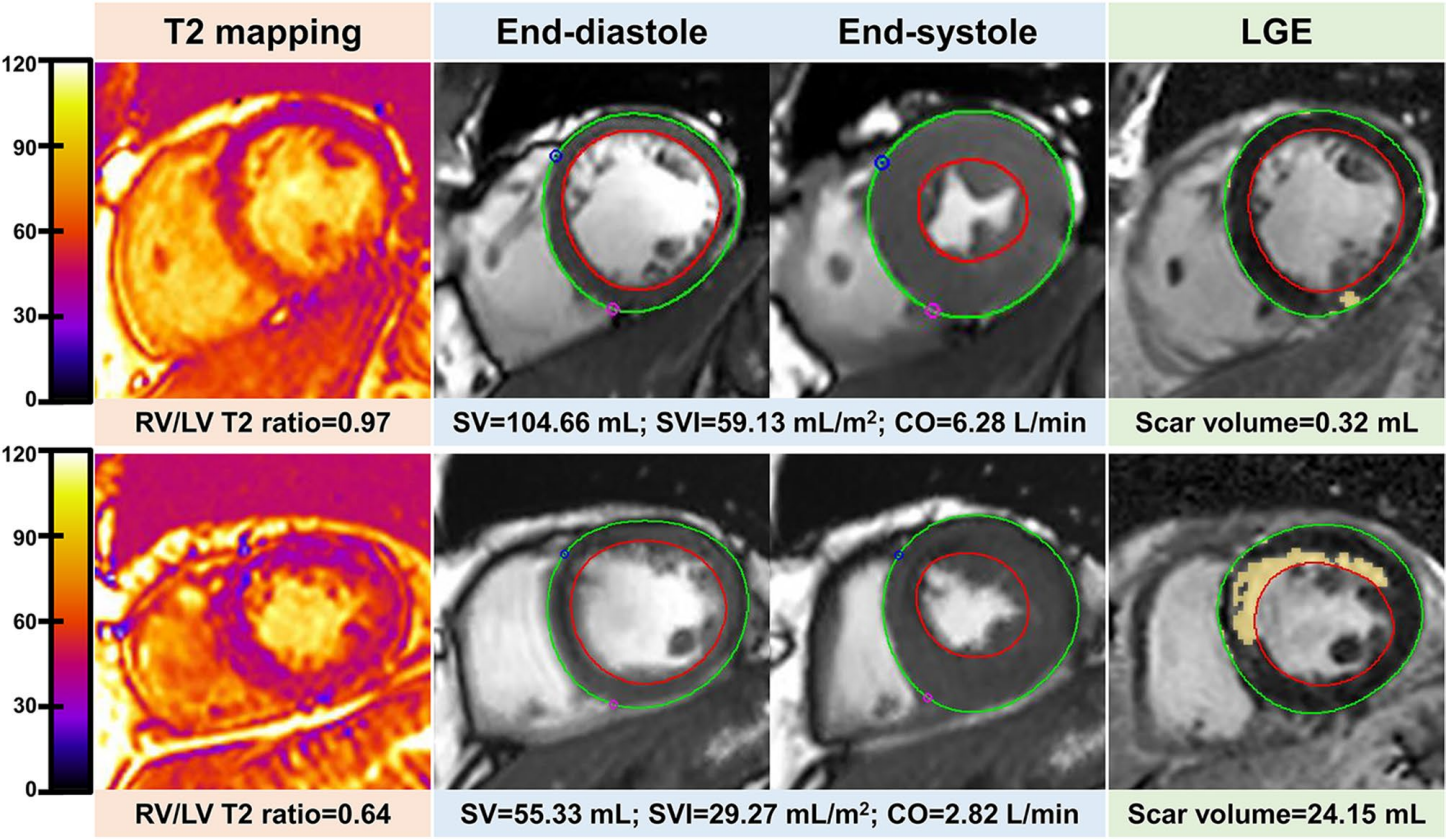
Representative short-axis T2 map, cine, and LGE images from a 56-year-old man with MMSE score of 29 and MoCA score of 26 (upper panel), and a 69-year-old man with MMSE score of 28 and MoCA score of 20 (lower panel). LGE, late gadolinium enhancement; MMSE, Mini-Mental State Exam; MoCA, Montreal Cognitive Assessment; RV, right ventricle; LV, left ventricle

There were positive associations of RV/LV T2 ratio with CMR parameters including SV (*r* = 0.569, *p* < 0.001) (Fig. 2C), SVI (*r* = 0.518, *p* < 0.001) (Fig. 2D), CO (*r* = 0.504, *p* < 0.001) (Fig. 2E), and CI (*r* = 0.410, *p* = 0.003) (Fig. 2F). Figure 3 presents representative CMR images from two patients with normal and decreased MoCA score.

**Fig. 4:**
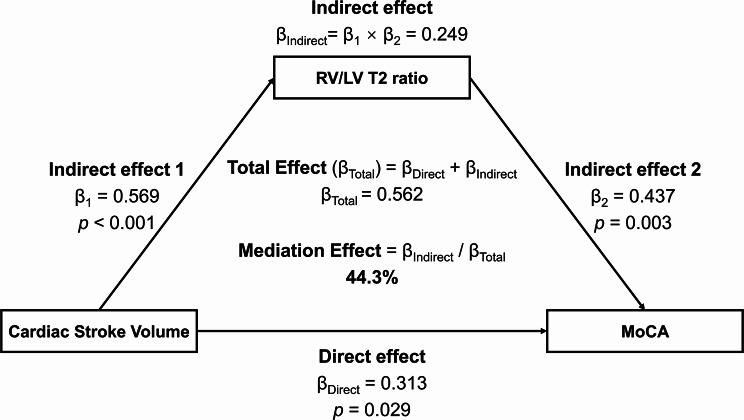
Mediation effect of RV/LV T2 ratio on the association between cardiac stroke volume and cognitive performance, adjusting for age, sex, and education. β, standardized coefficient; β_1_, indirect effect 1; β_2_, indirect effect 2; β_Indirect_, total indirect effect; β_Direct_, direct effect; β_Total_, total effect; RV, right ventricle; LV, left ventricle; MoCA, Montreal Cognitive Assessment

Mediation effect of RV/LV T2 ratio on SV and MoCA score was evaluated with mediation analysis, with SV, RV/LV T2 ratio, and MoCA as the independent variable, mediating variable, and dependent variable, respectively. The results demonstrated that RV/LV T2 ratio partially mediated the correlation between SV and MoCA score (indirect effect standardized coefficient β = 0.249) after adjustment for age, sex, and education, with a mediation effect ratio of 44.3% (Fig. 4).

### Reproducibility analysis

**Fig. 5:**
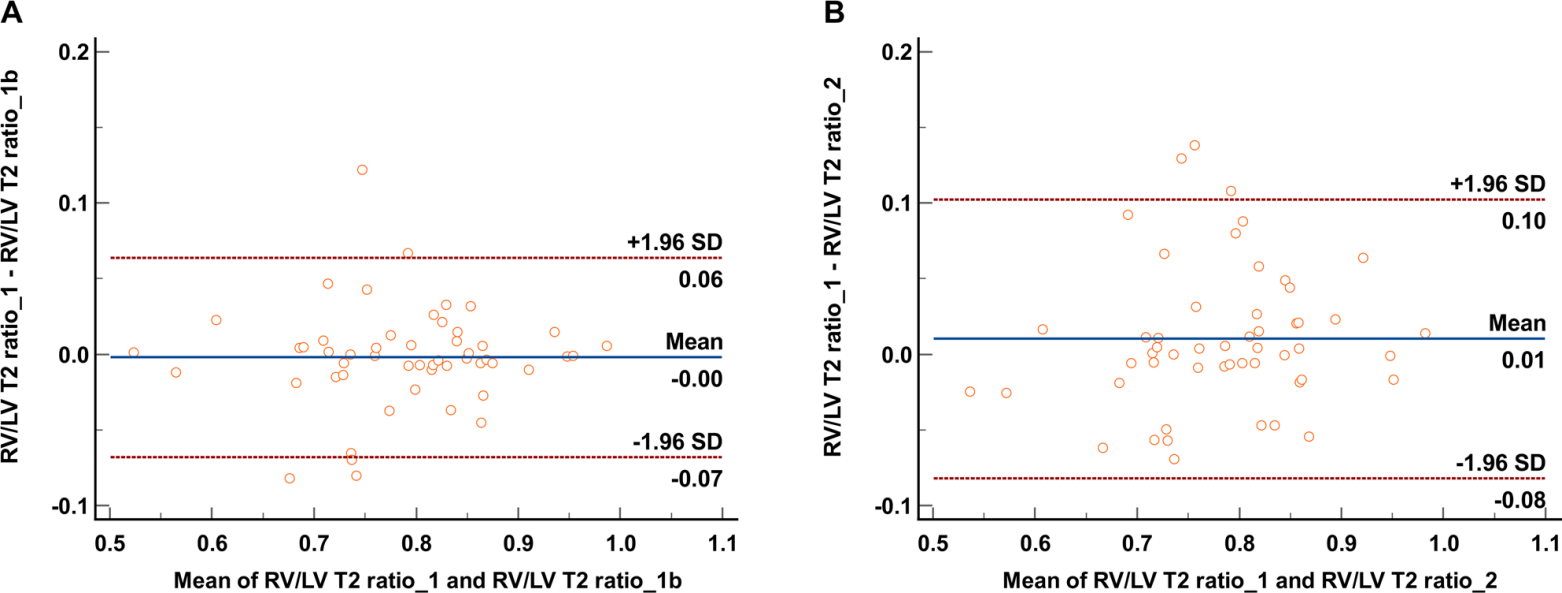
Bland-Altman plots of intra-observer (**A**) and inter-observer (**B**) RV/LV T2 measurements in patients with HF. RV, right ventricle; LV, left ventricle

Excellent intra-observer agreement (ICC = 0.936, 95% confidence interval (CI) 0.891–0.963) and good inter-observer (ICC = 0.870, 95% CI 0.785–0.923) agreement were observed for RV/LV T2 ratio measurements. Bland-Altman plots of RV/LV T2 ratio are presented in Fig. 5. The intra-observer mean difference and 95% limits of agreement was − 0.002 (95% CI -0.011-0.007) and − 0.07 to 0.06. The inter-observer mean difference and 95% limits of agreement was − 0.010 (95% CI -0.003-0.023) and − 0.08 to 0.10.

## Discussion

The present study investigated RV/LV T2 ratio and its relationship with cognitive performance in patients with HF secondary to IHD. The key findings were as follows: RV/LV T2 ratio was lower in patients with HF than HC. Among HF patients, those with cognitive impairment (MoCA-defined) exhibited a markedly reduced RV/LV T2 ratio relative to those with normal cognition. In addition, RV/LV T2 ratio was positively correlated with cognitive performance. Multivariable regression analysis revealed that, among all CMR parameters, RV/LV T2 ratio was the only independent predictor for MoCA score. Subsequent mediation analysis confirmed that RV/LV T2 ratio partially mediated the correlation between SV and MoCA score, suggesting circulatory hypoxia resulting from systemic hypoperfusion is a potential contributor to cognitive impairment in HF secondary to IHD.

Increasing evidence indicated cognitive impairment and dementia in HF patients was correlated with poor prognosis [3]. Cerebral hypoperfusion was proposed as a critical underlying mechanism involved in the development of cognitive impairment [26]. In line with several large community-based cohort studies [27–29], our findings demonstrated a positive association between SV and cognitive performance, suggesting that reduced systemic perfusion may contribute to worse cognitive function. Reduced systemic blood flow is correlated with decreased cerebral blood flow (CBF) [30]. Long-term cerebral hypoperfusion and disruption of cerebral circulatory homeostasis may contribute to brain tissue alterations including ischemic subcortical whiter matter hyperintensities and gray matter loss [27, 31]. Consistent with observations in normal aging populations, prior neuroimaging studies in HF patients have identified various brain abnormalities using diverse MRI modalities, including gray matter loss on structural MRI [32, 33], breakdown of axonal integrity on diffusion tensor imaging (DTI) [34], aberrant functional connections on resting-state functional MRI [35], and cerebral hypoperfusion on arterial spinal labeling (ASL) [5]. Besides, recent study demonstrated cerebral metabolism on positron emission tomography (PET) partially mediated the relationship between SV and cognitive function [36].

T2 mapping is a unique technique for noninvasive assessment of myocardial edema in a broad range of pathological situations and has become an essential component of comprehensive CMR protocol [37]. For the blood, the oxygenation state of hemoglobin could affect transverse relaxation. Therefore, blood T2 time is dependent on oxygen saturation and has been successfully utilized in quantification of blood oxygenation level. However, previous methods required investigational sequence, additional imaging acquisition, internal calibrations, and complex post-processing approach, hampering its clinical applications [15, 38, 39]. In contrast, RV/LV T2 ratio is based on conventional T2 mapping and could be easily performed by ROI analysis of the blood pool. Although RV/LV T2 ratio cannot directly quantify the blood oxygenation level, it may provide complementary information about the oxygen saturation difference between RV and LV to standard CMR evaluation. This method was first proposed by Emrich et al. and applied to diagnose left-to-right shunts and assess shunt severity [17]. Besides, it was also found to be correlated with blood oxygen saturation measured via right heart catheterization in patients with pulmonary hypertension, and helpful to evaluate the disease severity and therapy response [18, 19]. Recent study reported a positive relationship between RV/LV T2 ratio and exercise capacity in HF patients [20]. Our study extended earlier work by demonstrating the relationship between reduced venous and arterial blood oxygen saturation difference determined by RV/LV T2 ratio and impaired cognitive performance. Thus, RV/LV T2 ratio shows potential as a simple and noninvasive imaging method for detection of cognitive impairment in HF patients.

A notable finding in our study is that RV/LV T2 ratio differed significantly between cognitively normal patients and those with cognitive impairment when assessed using MoCA score, whereas no statistically significant difference was observed based on MMSE score. Additionally, although univariate linear regression analysis indicated a correlation between RV/LV T2 ratio and both MoCA and MMSE scores, this association was not significant for MMSE score in the multivariate linear regression analysis. Two potential explanations may account for these results. First, this may be attributed to the relatively small sample size, leading to reduced statistical power. Specifically, only 10 patients were diagnosed with cognitive impairment based on MMSE score, which may lead to insufficient statistical power to detect the difference in RV/LV T2 ratio. Besides, the correlation between RV/LV T2 ratio and MMSE score observed in univariate analysis may not be detectable in multivariate analysis, as reduced statistical power hinders the identification of weak association. Therefore, the association between RV/LV T2 ratio and MMSE requires further validation in larger cohort studies. Second, these results also demonstrate that MoCA is more comprehensive and sensitive in detecting cognitive impairment and appears to be a better screening tool compared to MMSE in patients with HF [40, 41].

As systemic circulation is usually reduced in chronic HF, oxygen dissociation curve shifts to the right with compensatory decrease of hemoglobin oxygen affinity, resulting in an increase of oxygen delivery to the peripheral tissues [42]. Lower RV/LV T2 ratio generally reflects greater peripheral tissue oxygen extraction in chronic HF. Due to its high energy demands, the brain is especially vulnerable to hypoxia. Thus, circulatory hypoxia in chronic HF may be generalized, resulting in cerebral hypoxia, brain injury, and subsequent cognitive impairment, which may explain the association between RV/LV T2 ratio and cognitive performance [43]. We also found that CO was positively related to RV/LV T2 ratio, indicating lower CO was associated with higher difference between venous and arterial oxygen saturation, which was in accordance with the Fick principle [44]. Similarly, CI was reported to be associated with RV T2 value in pulmonary hypertension [19]. Therefore, RV/LV T2 ratio may serve as a biomarker of circulatory hypoxia and cardiac hemodynamics. Previous research reported lower RV/LV T2 ratio in HF patients with higher NYHA classes, suggesting RV/LV T2 ratio was related to the severity of HF [20]. In the multivariate regression analysis of the present study, the association between NYHA class and cognitive scores became nonsignificant after adjusting for multiple covariates, whereas the correlation between RV/LV T2 ratio and cognitive scores remained significant. These findings indicate that the RV/LV T2 ratio is not merely a surrogate for the severity of HF, but may indeed serve as a biomarker for cognitive impairment.

Absolute T2 relaxation time measurement is determined by various factors, such as manufacturer, field strength, imaging sequence, as well as imaging parameters [38]. RV/LV ratio approach seems more reproducible under different imaging conditions. In addition to hemoglobin and oxygen saturation, blood pool T2 values are also partly influenced by hematocrit and serum iron [22]. Nevertheless, the association between RV/LV T2 ratio and these factors remains unclear and requires further investigation. The value of RV/LV T2 ratio we reported was comparable to previous studies [17–19]. Nevertheless, another study conducted in chronic HF patients reported a much lower RV/LV T2 ratio compared to the present results [20]. The exact explanation for this discrepancy is unclear, but might be attributed to clinical characteristics, severity, and etiologies of HF. Besides, ROI placement on different imaging planes may also affect the results [18]. Therefore, additional research is required to identify the potential influencing factors of T2 ratio measurement and establish a standardized analysis method.

### Limitation

Several limitations of this study should be acknowledged. First, this was a single-center study and the sample size in each subgroup was relatively small. The retrospective design may introduce selection bias and unmeasured confounding factors. Besides, this cross-sectional study cannot establish causality. Additional longitudinal study with larger samples would be required to determine the predictive value of RV/LV T2 ratio on cognition decline in HF patients. Second, the routine T2 mapping sequence was designed to evaluate the myocardium, but not the blood pool. It is sensitive to flow turbulence and magnetic field inhomogeneity, resulting in high variability of blood pool T2 measurements in some cases. Therefore, ROI should be carefully placed to avoid flow artifact. In the present study, measurements from three short-axis slices were averaged to minimize the variability. Third, this study only included patients with HF secondary to IHD, therefore, the present results should be verified in HF patients with other etiologies.

## Conclusion

RV/LV T2 ratio is a simple and noninvasive CMR biomarker that may serve as a promising indicator for cognitive impairment in patients with HF secondary to IHD. Additional research is needed to elucidate the potential role of RV/LV T2 ratio in the management and prognosis prediction of HF patients.

## Data Availability

The datasets generated and/or analyzed during the current study are not publicly available due to patient privacy and institutional restrictions, but are available from the corresponding author on reasonable request and with appropriate ethical approval.
